# Gene promoter hypermethylation in ductal lavage fluid from healthy *BRCA *gene mutation carriers and mutation-negative controls

**DOI:** 10.1186/bcr1657

**Published:** 2007-02-26

**Authors:** Imogen Locke, Zsofia Kote-Jarai, Mary Jo Fackler, Elizabeth Bancroft, Peter Osin, Ashutosh Nerurkar, Louise Izatt, Gabriella Pichert, Gerald PH Gui, Rosalind A Eeles

**Affiliations:** 1 Translational Cancer Genetics Team, Institute of Cancer Research, 15 Cotswold Road, Sutton, Surrey SM2 5NG, UK; 2 Department of Cancer Genetics, Royal Marsden NHS Foundation Trust, Fulham Road, London SW3 6JJ, UK; 3 Department of Oncology, Johns Hopkins University School of Medicine, 401 North Broadway, Baltimore, MD 21231, USA; 4 Department of Pathology, Royal Marsden NHS Foundation Trust, Fulham Road, London SW3 6JJ, UK; 5 Department of Genetics, Guy's and St Thomas' NHS Foundation Trust, St Thomas Street, London SE1 9RT, UK; 6 Department of Surgery, Royal Marsden NHS Foundation Trust, Fulham Road, London SW3 6JJ, UK

## Abstract

**Introduction:**

Female germline *BRCA *gene mutation carriers are at increased risk for developing breast cancer. The purpose of our study was to establish whether healthy *BRCA *mutation carriers demonstrate an increased frequency of aberrant gene promoter hypermethylation in ductal lavage (DL) fluid, compared with predictive genetic test negative controls**, **that might serve as a surrogate marker of *BRCA1/2 *mutation status and/or breast cancer risk.

**Methods:**

The pattern of CpG island hypermethylation within the promoter region of a panel of four genes (*RAR-β*, *HIN-1*, *Twist *and *Cyclin D2*) was assessed by methylation-specific polymerase chain reaction using free DNA extracted from DL fluid.

**Results:**

Fifty-one DL samples from 24 healthy women of known *BRCA *mutation status (7 *BRCA1 *mutation carriers, 12 *BRCA2 *mutation carriers and 5 controls) were available for methylation analysis. Eight of 19 (42.1%) *BRCA *mutation carriers were found to have at least one hypermethylated gene in the four-gene panel. Two *BRCA *mutation carriers, in whom aberrant methylation was found, also had duct epithelial cell atypia identified. No hypermethylation was found in DL samples from 5 negative controls(*p *= 0.13).

**Conclusion:**

We found substantial levels of aberrant methylation, with the use of a four-gene panel, in the fluid from the breasts of healthy *BRCA *mutation carriers compared with controls. Methylation analysis of free DNA in DL fluid may offer a useful surrogate marker for *BRCA1/2 *mutation status and/or breast cancer risk. Further studies are required for the evaluation of the specificity and predictive value of aberrant methylation in DL fluid for future breast cancer development in *BRCA1/2 *mutation carriers.

## Introduction

Women carrying pathogenic gene mutations in either *BRCA1 *or *BRCA2 *are at significantly increased lifetime risk of up to 80% for developing breast cancer [1]. A significant proportion of this risk occurs in women under the age of 50 years. Current surveillance recommendations include mammographic screening and clinical breast examination [2]. It is well recognised that mammograms are less sensitive in younger women, who have more radiodense breast tissue, and although alternative imaging modalities such as magnetic resonance imaging have shown promise there is still a clear need for better risk assessment and earlier breast cancer detection in this high-risk group [3,4]. Ductal lavage (DL) is a novel method for repeated minimally invasive sampling of breast ductal fluid, allowing the safe collection of cells sufficient for cytological diagnosis and providing a source of cellular and free DNA for molecular analyses [5]. The predictive value of breast epithelial cell atypia, identified by DL, for breast cancer development is currently being assessed in the ongoing multicentre SEDE (Serial Evaluation of Ductal Epithelium) trial in women with moderate and high risk for breast cancer on the basis of family history criteria.

Over 60 women from known *BRCA *gene mutation carrying families are taking part in the ductal research programme at the Royal Marsden NHS Foundation Trust, which is evaluating the usefulness of nipple aspiration (NA) and DL as risk assessment tools in this group. We are using DL to investigate epithelial cell atypia rates among *BRCA *mutation carriers and are performing a variety of molecular and proteomic analyses on the ductal fluid collected in the search for surrogate biomarkers of breast cancer risk.

CpG islands are short regions of DNA containing clusters of CpG dinucleotides that are generally unmethylated in normal somatic cells. Hypermethylation of cytosine residues in CpG islands within the gene promoter is recognised as an important epigenetic mechanism of transcriptional silencing during early cancer development [6]. Key targets of aberrant promoter hypermethylation in breast cancer development include genes involved in all stages of tumorigenesis such as DNA repair (*BRCA1*), receptors (*ER*, *RAR-β*), intracellular signalling pathways, cell cycle regulation (*Cyclin D2*, *p16*^*INK4A*^), transcription factors (*Twist*), adhesion molecules (*E-cadherin*) and apoptosis (*HOXA5*) [7-14]. Gene promoter hypermethylation of *RAR-β*, *HIN-1*, *Cyclin D2 *and *Twist *has been reported to be a frequent and tumour specific event in *in situ *and invasive breast cancer of both ductal and lobular types [10]. In this study we sought to determine whether there was an association between hypermethylation of four candidate tumour suppressor genes, implicated in breast carcinogenesis, and underlying *BRCA *gene mutation status.

The observation that levels of cell-free DNA are higher in the body fluids of cancer patients than in healthy controls has led to interest in its use in the screening and early diagnosis of cancer [15]. Cancer-specific DNA methylation patterns have been found in exfoliated luminal tumour cells and free tumour DNA from a variety of body fluids including urinary sediment, saliva, sputum, bronchial washings and ejaculate [16-21]. Previous studies have reported the methylation patterns of cellular DNA from nipple aspirates and DL fluid obtained from women with breast cancer compared with those with benign breast disease and healthy controls. The use of free DNA from DL fluid for methylation profiling is novel [22,23].

Methylation-specific PCR (MSP) requires only small quantities of DNA, has high specificity and is sensitive enough to identify one methylated allele among 1,000 unmethylated alleles [24]. Aberrant hypermethylation of CpG islands, being uncommon in normal cells and an early event in cancer development, is a good candidate for a biomarker of breast cancer risk. Breast ductal fluid can be repeatedly sampled in a minimally invasive way, and methylation analysis, in conjunction with cytological diagnosis, potentially offers a further tool for assessing individual risk for developing breast cancer.

## Materials and methods

### Subjects

Prospective locoregional ethics committee approval was gained for a study evaluating the usefulness of DL as a breast cancer risk assessment tool in women from families carrying the *BRCA1/2 *gene mutation who attended the Cancer Genetics Carrier Clinic at the Royal Marsden Foundation NHS Trust. This ongoing study is comparing breast ductal epithelial cell atypia rates in the lavage fluid of *BRCA *gene mutation carriers with true negative controls (women who have had a negative predictive genetic test for a known *BRCA1 *or *BRCA2 *mutation present in the family). All women had had a normal clinical breast examination and mammogram within 12 months of the sample collection and gave informed consent before entry to the study. DL was attempted on all healthy breasts unaffected by previous cancer; where possible, multiple ducts in the same breast were cannulated. Fifty-one DL specimens were available for methylation pattern analysis from 24 women, of whom 19 were *BRCA *mutation carriers (7 *BRCA1 *and 12 *BRCA2*) and 5 were negative controls on the basis of the predictive genetic test. Eight of the 51 DL samples were repeat samples from individual ducts, identified by marking the duct position on a grid, from 6 *BRCA *mutation carriers collected 1 year apart. Five of the *BRCA2 *mutation carriers had previously been affected by unilateral breast cancer (three with invasive ductal carcinoma, two with invasive lobular cancer) but were currently disease free.

### Specimen collection and processing

Topical anaesthetic cream was applied to the nipple and areola 1 hour before DL was performed, and 0.5 to 1.0 ml of 1% lignocaine was injected subcutaneously at the base of the nipple as this was found to improve tolerability. DL was otherwise performed as described by Dooley and colleagues with the modification that lavage was attempted of both nipple aspirate fluid-yielding and non fluid-yielding ducts [5]. The DL fluid was collected into 15 ml Falcon tubes and then centrifuged at 1,500 r.p.m. for 10 minutes at 4°C. Two slides, produced using the Shandon cytospin technique from the cellular fraction of the DL sample, were Giemsa-stained for cytological assessment. Slides were deemed adequate for cytological diagnosis if more than 10 epithelial cells were present. Slides with fewer than 10 epithelial cells were considered to contain inadequate cellular material for diagnosis (ICMD). Slides with sufficient epithelial cells for diagnosis were further categorised as benign ductal epithelial cells, mildly atypical epithelial cells, markedly atypical cells or malignant epithelial cells. Cytological assessment was performed by breast cytopathologists (PO and AN) blinded to the genetic status of the subject. The DL supernatant was immediately divided into aliquots and stored at -80°C for later DNA extraction. Free DNA was extracted from 200 μl of DL supernatant, using the DNeasy Tissue Kit (Qiagen, Hilden, Germany) in accordance with the manufacturer's instructions, and eluted in 50 μl of the AE buffer provided in the kit. Unmethylated control samples consisted of 1 mg/ml solutions of human sperm DNA (HSD), and methylated controls were 1 mg/ml solutions of DNA extracted from the MDA-MB-231 breast cancer cell line (231), both provided courtesy of Mary Jo Fackler (Johns Hopkins University School of Medicine, Baltimore, MD, USA).

### Sodium bisulphite conversion

Sodium bisulphite and alkaline treatment of genomic DNA converts unmethylated cytosine residues to uracil, leaving methylated cytosine residues unchanged. These methylation-dependent sequence variants at a specific locus can subsequently be analysed by PCR amplification by using primers specific for the unmethylated or methylated sequence. A modification of the method described by Herman and colleagues was used for the sodium bisulphite conversion of both DL supernatant DNA samples and the unmethylated/methylated control samples [24]. In brief, either 13.5 μl of the DL supernatant DNA solution containing 600 μg of salmon sperm DNA carrier or 1 μl control DNA with 12.5 μl TNES (10 mM Tris-HCl pH 8.0, 150 mM NaCl, 2 mM EDTA, 0.5% SDS) containing 830 ng of salmon sperm DNA was heated to 99°C for 10 minutes. After the addition of 1.5 μl of 2 M NaOH, the sample was incubated at 42°C for 30 minutes. Sodium bisulphite (3.6 M, 95 μl) containing 1 mM hydroquinone was added to each sample, which was then overlaid with three drops of mineral oil and incubated for 5 hours at 55°C in the dark. Two 50 μl aliquots taken from each of the sodium bisulphite modified DNA samples were applied separately to two S-200 Microspin columns (Amersham, Needham, MA, USA) and purified in accordance with the manufacturer's instructions. The DNA eluted from both columns was pooled in a 1.5 ml microtube with 10 μl of 3 M NaOH and chilled on ice for 5 minutes. Subsequently 275 μl of sterile water, 125 μl of 7.5 M ammonium acetate and 3 μl of glycogen were added to each sample and the mixture chilled for a further 5 minutes on ice. DNA was precipitated using 1 ml of absolute ethanol, washed in 1 ml of 75% ethanol and redissolved in 10 μl of water. The sample was stored at -80°C until use.

### Methylation analysis

Methylation analysis was performed with a two-stage PCR technique. An initial multiplex PCR reaction was performed to co-amplify template DNA in the promoter region of four genes (*RAR-β*, *HIN-1*, *TWIST *and *Cyclin D2*). Either 4 μl (DL samples) or 1 μl (HSD/231 controls) of the purified sodium bisulphite converted DNA solution were included in a 25 μl PCR reaction buffer (16.6 mM (NH_4_)_2_SO_4_, 67 mM Tris-HCl pH 8.8, 6.7 mM MgCl_2_, 10 mM 2-mercaptoethanol, 0.1% dimethylsulphoxide, 1.25 mM dNTP mixture) also containing 5 units of Platinum *Taq *(Invitrogen, Carlsbad, CA, USA) and 100 ng each of the forward and reverse primers specific for the four-gene panel of interest. Primer sequences and PCR conditions for the multiplex step were as described by Fackler and colleagues [25]. The PCR products were diluted to a total volume of 125 μl with water and stored at -20°C. The HSD and 231 controls were further diluted 1:1,000 with water.

The second stage was a methylation-specific PCR reaction. Diluted DNA (1 μl) from the multiplex PCR reaction was added to a 24 μl PCR reaction buffer described above containing 2.5 units of RedTaq (Sigma, St Louis, MO, USA) and 100 ng each of the forward and reverse primers specific for either the unmethylated or methylated variants of the gene of interest after sodium bisulphite conversion. All primer sets were obtained from Invitrogen, and the sequences have been reported previously [10]. PCR products were separated on a 2% agarose electrophoresis gel. Replicate MSP reactions were performed at least twice to ensure reproducibility of results.

### Statistical analysis

Fisher's exact test (two-sided) was used to compare the proportion of *BRCA *mutation carriers and controls with hypermethylation in their DL samples.

## Results

Fifty-one DL samples from 24 healthy women of known *BRCA *status (7 *BRCA1 *carriers, 12 *BRCA2 *carriers and 5 controls) were available for methylation analysis. A mean of 1.2 ducts were successfully cannulated per breast on each visit. The mean ages of *BRCA *mutation carriers and controls at their first DL sample collection were 44.9 years (range 34.3 to 62.8 years) and 50.4 years (range 41.4 to 55.4 years), respectively. Thirty-eight of the 51 DL samples contained adequate cells for cytological diagnosis (74.5%). Thirty-six of the 51 DL samples demonstrated benign cytology (70.6%) and two samples from *BRCA *mutation carriers demonstrated mild atypia (3.9%). A further 13 of the 51 samples were classified as ICMD. No atypia was identified in the DL samples from predictive genetic test negative controls.

**Figure 1:**
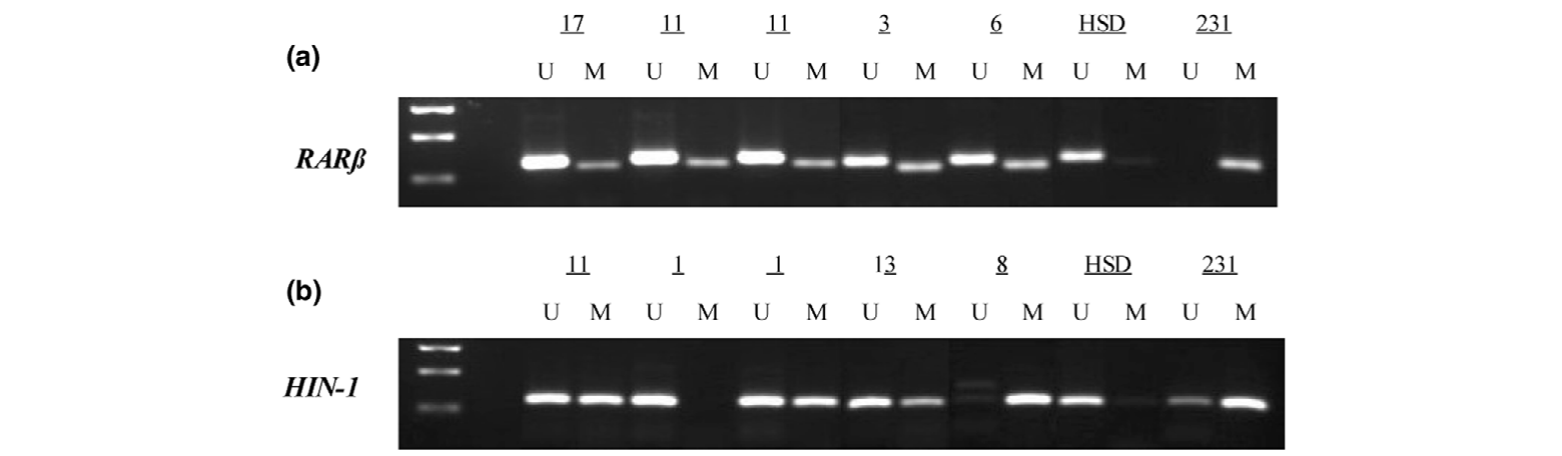
Representative methylation analysis of DL samples from *BRCA *carriers in whom aberrant methylation was found. **(a) **Hypermethylation of *RAR-β *found in ductal lavage (DL) samples from subjects 17, 11, 3 and 6. Replicate PCR experiments for subject 11, using DNA from the same duct, are shown to illustrate reproducibility. **(b) **Methylation analysis of *HIN-1*. Two different ducts for subject 1 are shown: the first is unmethylated (duct right 2a; see Figure 2) and the second shows hypermethylation of *HIN-1 *(duct right 1a). Subjects 11, 13 and 8 show aberrant methylation of HIN-1. In both panels, unmethylated control samples consisted of 1 mg/ml solutions of human sperm DNA (HSD), and methylated controls were 1 mg/ml solutions of DNA extracted from the MDA-MB-231 breast cancer cell line (231). Marker used is a 123bp DNA ladder - rungs represent 123bp, 246bp and 369bp. M, methylated; U, unmethylated.

**Figure 2:**
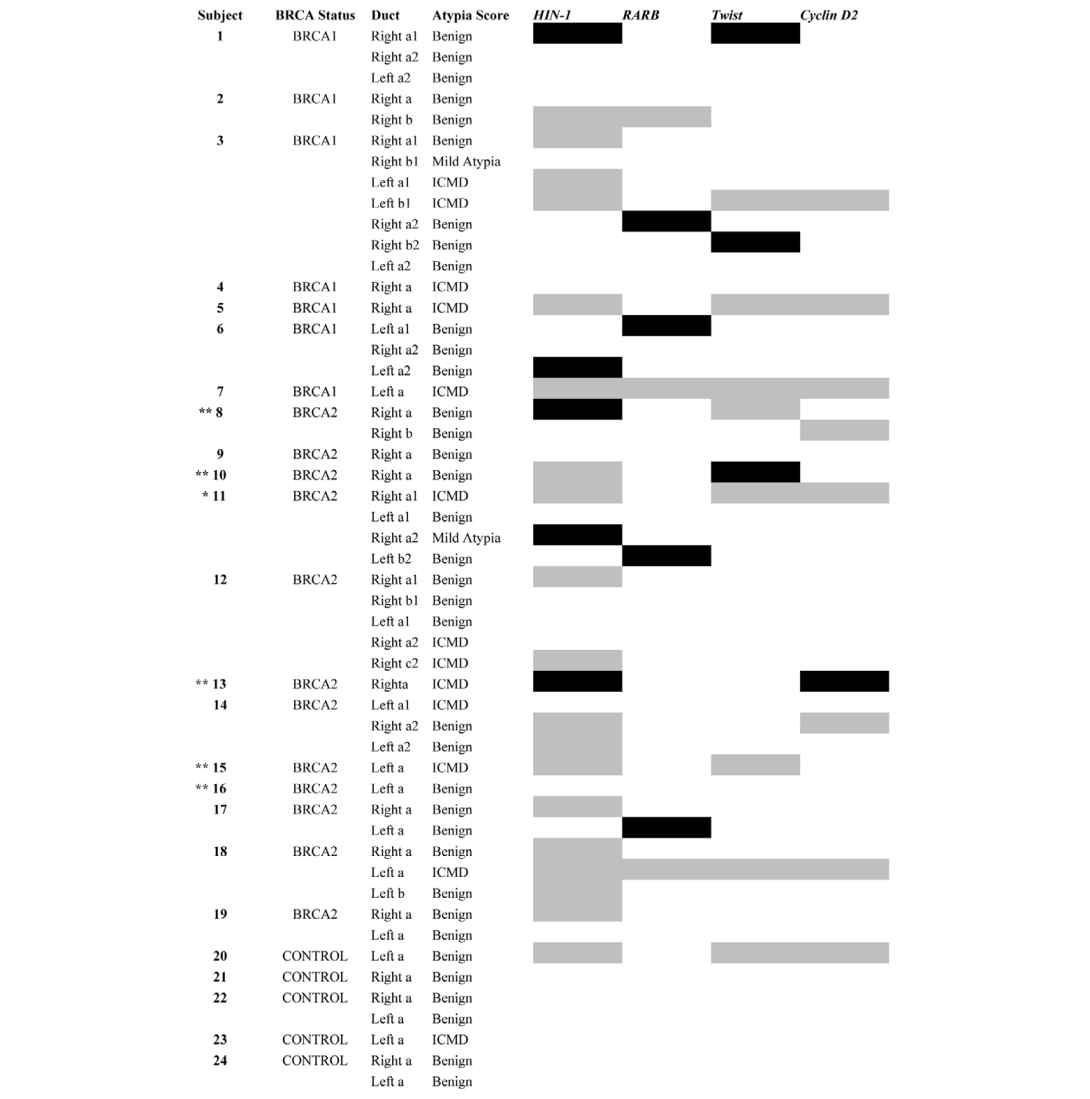
Methylation analysis of four-gene panel in DL fluid from *BRCA1 *and *BRCA2 *carriers and controls. The genes in the panel were *HIN-1*, *RAR-β*, *Twist *and *Cyclin D2*. Ducts are identified as being from the left or right breast. Individual ducts are identified sequentially as a, b or c. The suffix 1 or 2 indicates the first or second lavage visit, respectively, for ducts from which repeat samples were taken 1 year apart. For example, left a2 indicates that a sample was taken from the left breast, duct a at the second ductal lavage (DL) visit. Black boxes indicate methylated genes, white boxes indicate unmethylated genes, and grey boxes indicate repeated failure of PCR reaction or inconclusive result. *Subject 11 had long-standing atypia identified in both breasts on nipple aspirate fluid cytology. **BRCA2 carriers affected by contralateral breast cancer. DL samples were collected from only their healthy unaffected breasts. ICMD, insufficient cellular material for diagnosis.

Free DNA sufficient for PCR amplification was obtained from the supernatant of 49 of 51 DL samples. Eight of 19 (42.1%) mutation carriers were found to have at least one hypermethylated gene in the four-gene panel, in comparison with none of the 7 DL samples obtained from 5 negative controls (*p *= 0.13). We found *HIN-1 *to be the most frequently methylated gene and *CyclinD2 *the least frequently methylated gene in the panel (Table 1). Representative examples of methylated DL samples are shown in Figure 1. Four of the eight *BRCA *mutation carriers, in whom aberrant methylation was found, demonstrated simultaneous methylation of two different genes in the four-gene panel – two *BRCA *mutation carriers in the same DL sample (subjects 1 and 13) and two *BRCA *mutation carriers in different ducts (subjects 3 and 11). A further *BRCA *mutation carrier (subject 6) was found to have hypermethylation of *RAR-β *in a DL sample from the left breast at her first visit and hypermethylation of *HIN-1 *in a repeat sample taken from the same duct 1 year later (Figure 2).

Two *BRCA *mutation carriers, in whom aberrant methylation was demonstrated, also had asymptomatic duct epithelial cell atypia. The first, a *BRCA1 *mutation carrier (subject 3), had mild atypia identified in a DL sample taken from the right breast at her initial DL visit. Although aberrant methylation was not identified in the DL samples taken at this time, hypermethylation of *RAR-β *and *Twist *was subsequently found in DL samples taken from the same breast 1 year later. The second, a *BRCA2 *mutation carrier (subject 11), was known to have persistent mild atypia in both breasts on repeat nipple aspirate cytology in a previous study. Mild atypia was identified in a DL sample taken from the right breast, and MSP analysis of DNA extracted from the same duct revealed hypermethylation of *HIN-1*. At the same visit hypermethylation of *RAR-β *was found in a DL sample taken from the left breast although on this occasion DL cytology was benign.

Clearly, reproducibility is an important issue if hypermethylation is to be a useful biomarker of breast cancer risk, and replicate PCR experiments showed our assay to be robust. We examined reproducibility of the methylation analysis over time, in a subgroup of women, by repeat sampling of DL fluid from individual marked ducts 1 year apart. We found that the presence of methylation was not always consistent but that in most ducts the change in methylation status was from unmethylated to methylated, which could represent an alteration in risk over time. There was no statistically significant association between age and the presence of hypermethylation in *BRCA *mutation carriers. Hypermethylation was more commonly found in premenopausal (6 of 12 women) than postmenopausal (2 of 7 women) *BRCA *mutation carriers, but this did not reach statistical significance.

Three of the five *BRCA2 *mutation carriers previously affected by contralateral breast cancer were found to have aberrant methylation in DL samples from their healthy breasts. Of those previously affected by contralateral invasive ductal carcinoma, two were found have hypermethylation of *HIN-1 *and one also had hypermethylation of *Cyclin D2*. One of the two DL samples from women with previous invasive lobular carcinoma demonstrated aberrant methylation of *Twist*. None of the women taking part in this study have been diagnosed with a new primary breast cancer during a median length of follow-up of 32 months (range 17 to 38 months).

We also examined whether hypermethylation was associated with the fluid-yielding status of the duct or DL sample cellularity. Large population-based studies of nipple aspirate cytology have shown that women who do not yield NA fluid have the lowest risk for developing breast cancer and that women producing NA fluid with benign cytology have a relative risk for developing breast cancer of 1.2 to 1.6 compared with those who do not yield NA fluid [26]. Recent studies of DL in *BRCA1/2 *mutation carriers have examined the influence of various hormonal factors on fluid-yielding status and DL sample cellularity. Younger age and premenopausal status are predictive of higher DL fluid yields and increased sample cellularity [27,28]. Furthermore, an inverse correlation exists between fluid-yielding status and previous treatment for breast or ovarian cancer with therapies likely to impair ovarian function, suggesting the importance of oestrogen in maintaining the proliferative status of the breast ductal epithelium [27].

In our study, of the 44 DL samples from *BRCA *mutation carriers, 34 samples were from fluid-yielding ducts and 10 from ducts not yielding fluid. Interestingly, all 11 DL samples demonstrating aberrant methylation were from ducts that yielded fluid with nipple suction aspiration. Methylation was also found more commonly in cellular DL samples than in samples that were ICMD. However, the predictive value of fluid-yielding status and cellular atypia in DL fluid for breast cancer development remains unproven in prospective trials. Indeed, some studies have reported similar rates of DL atypia for both fluid-yielding ducts and those not yielding fluid, suggesting that dry ducts may carry a higher risk of breast cancer than previously thought [27,29].

## Discussion

Women carrying a germline heterozygous mutation in either *BRCA1 *or *BRCA2 *are predisposed to breast and ovarian cancer. DL has been shown to be a safe and feasible method for retrieving breast ductal epithelial cells and sampling their surrounding ductal microenvironment in a minimally invasive way [5]. Studies have shown early promise in identifying potential markers of breast cancer risk in ductal fluid, but validation of these potential molecular markers and further evaluation of the ductal approach to establish the sensitivity and specificity for early breast cancer detection is required [22,30,31]. DL cytology alone has been found to have a low sensitivity for the detection of established breast cancer, but this may be improved by markers such as gene promoter hypermethylation [32,33].

Hypermethylation of gene promoter regions is an early event in breast carcinogenesis; cancer-specific DNA methylation profiles of free tumour DNA in blood and other body fluids have been reported, but the diagnostic potential of DNA methylation profiling remains largely unexplored [28]. NA and DL offer the potential for repeatedly collecting breast epithelial cells and ductal fluid, providing a source of both cellular and free DNA for methylation studies. Krassenstein and colleagues studied the methylation patterns of a six-gene panel (*GSTP1*, *RAR-β*, *RASSF1A*, *DAP-kinase*, *p16*^*INK4a *^and *p14*^*ARF*^) in 22 paired breast tumour and nipple aspirate fluid (NAF) DNA samples [23]. Hypermethylation of at least one gene was present in all 22 of the tumour samples, and in 18 (82%) of the matched nipple aspirate specimens the same genes were methylated. However, not all women produce NAF and in our experience only about 40% of *BRCA *mutation carriers are fluid yielders (Locke I, unpublished data). This figure is similar to that reported in a previous study of NA and DL in *BRCA *mutation carriers and may reflect the increasing use of risk reduction strategies, such as bilateral prophylactic oophorectomy and chemopreventative agents, which are associated with a reduced rate of NAF production and consequently limit the utility of methylation profiling of NAF in this group as a risk assessment tool [27,34].

DL has the advantage of providing a more cellular yield than NAF; furthermore DL is often possible in non-NAF producers, potentially making it a more clinically useful source of DNA for methylation studies. Evron and colleagues [22] reported that methylation analysis, with a three-gene marker panel (*Cyclin D2*, *RAR-β *and *Twist*), of DNA from the exfoliated cells in 56 DL samples from asymptomatic women at increased risk for developing breast cancer (Gail risk at least 1.7-fold) had a sensitivity of 67% and a specificity of 89% for the detection of severely atypical or malignant epithelial cells. In our study we used free DNA extracted from the DL supernatant to allow the relatively limited cellular component of the DL sample to be conserved for cytological assessment and other molecular studies.

Free DNA from DL supernatant has previously been used to perform loss-of-heterozygosity analyses at the *BRCA1 *and *FHIT *loci and for the detection of mitochondrial DNA mutations in *BRCA1 *mutation carriers and controls, although questions have been raised about the robustness of these analyses [30,35,36]. The use of free DNA in DL fluid for methylation profiling has not previously been reported.

In our study, we assessed the methylation pattern of free DNA from DL fluid by using a small panel of genes implicated in early breast carcinogenesis. Retinoids are derivatives of vitamin A that bind to the RAR receptor and have chemopreventative potential mediated through their function in regulating cellular growth and differentiation. Retinoids have been found to inhibit the growth of breast cancer cells in culture and breast tumours in animal models [37]. Methylation of the *RAR-β *promoter region in breast cancer tumours and cell lines shows an inverse correlation with the degree of *RAR-β *gene expression; the gene is expressed and unmethylated in normal breast tissue and in human mammary epithelial cells [9,38]. Hypermethylation of the *RAR-β *promoter is a frequent event in both ductal and lobular breast cancer and has also been found to be correlated with the presence of macroscopic sentinel lymph node metastases, an important adverse prognostic factor [10,37].

*HIN-1 *is a putative cytokine, and promoter hypermethylation is thought to confer insensitivity to antigrowth signals. *HIN-1 *expression is downregulated in most breast cancers; reintroduction of *HIN-1 *into breast cancer cell lines inhibits cell growth, providing supporting evidence for its role as a candidate tumour suppressor gene [39]. Promoter hypermethylation of *Cyclin D2*, an important cell-cycle-regulatory gene that controls the transition from G1 to S phase, has been described in breast cancer. and its presence in ductal carcinoma *in situ *suggests that transcriptional silencing of *Cyclin D2 *by hypermethylation is an early event in breast tumorigenesis [11]. *Twist*, a basic helix – loop – helix transcription factor, has been implicated in cell lineage differentiation and the inhibition of oncogene-dependentand p53-dependent cell death. Promoter hypermethylation of *Twist *may permit cells to evade apoptosis and has been found to be a feature of both *in situ *and invasive breast cancer, particularly of the ductal rather than the lobular subtype [10,22].

We found *HIN-1 *and *RAR-β *to be more commonly hypermethylated than *CyclinD2 *or *Twist *in DNA from the DL fluid of *BRCA *mutation carriers. Interestingly, this finding is similar to that reported by previous authors for ductal carcinoma *in situ *and lobular carcinoma *in situ*, suggesting that hypermethylation of *HIN-1 *and *RAR-β *genes is a frequent and early event in breast carcinogenesis [10]. The four genes in the panel used in this study have pivotal roles in cell cycle regulation, the control of cell growth and p53-dependent apoptosis. Promoter hypermethylation and consequent transcriptional silencing of *HIN-1*, *RAR-β*, *Cyclin D2 *and *Twist *may remove the negative regulation of cell proliferation and apoptosis, allowing uncontrolled cell growth and evasion of cell death.

We have demonstrated that it is feasible to perform an MSP analysis for a panel of four genes with free DNA extracted from the DL supernatant of both *BRCA *germline mutation carriers and negative controls. Furthermore, it was possible to perform these analyses on acellular samples in which a cytological diagnosis is not possible, thus enhancing the potential diagnostic utility of the samples. We found evidence that methylation is a frequent event in the breasts of apparently healthy *BRCA *mutation carriers who are at high risk for developing breast cancer. Indeed, aberrant methylation was found in 42.1% of the *BRCA *mutation carriers but in none of five negative controls. Half of the *BRCA *mutation carriers in whom hypermethylation was found demonstrated the simultaneous methylation of two genes of the methylation panel. Although the small number of controls limited the ability of our study to reach statistical significance, the levels of methylation found among *BRCA *mutation carriers were higher than would be expected, because these genes are not commonly methylated in normal breast tissue [40]. A larger study is under way to validate the findings of this preliminary study.

DL is a technique that is prone to cell sampling variation, particularly when a relatively small atypical or malignant tumour cell population exists within an abundant mixed normal cell population. Similarly, the methylation pattern obtained from a single DL sample reflects the heterogeneity of DNA molecules sampled. Furthermore, the degree of CpG island methylation is not likely to be uniform: it varies between different cells and indeed between copies of the gene within the same cell. These factors may contribute to the variation in the methylation pattern of DL fluid between individual ducts and between samples taken from the same duct at different time points that we found in this study. Atypia and aberrant methylation may both reflect breast cancer risk status independently, and a combined approach using cytological diagnosis and the evaluation of DL methylation patterns offers the (as yet unproven) potential for refining the assessment of familial breast cancer risk. High-throughput and more sensitive quantitative methodologies for methylation profiling have been reported with the ability to detect a single methylated allele among 10^5 ^unmethylated copies [25]. These techniques would have particular application to the methylation analysis of free DNA in DL fluid, in which the DNA concentration is low. We plan to investigate hypermethylation in a larger DL sample set with a broader panel of genes.

## Conclusion

We conclude that performing methylation analyses of free DNA from DL supernatant is feasible, and in our study we found substantial levels of aberrant methylation in DL fluid from healthy *BRCA *gene mutation carriers in comparison with controls. Such epigenetic events may represent an early event in breast tumorigenesis, with methylation analysis of free DNA from DL fluid offering a useful alternative surrogate marker of breast cancer risk, particularly when samples are insufficient for cytological diagnosis, and/or they may be a marker of *BRCA1/2 *mutation status. Further larger studies with long-term follow-up are required for evaluating the specificity and predictive value of these methylated markers in DL fluid for the subsequent development of breast cancer.

## Abbreviations

DL = ductal lavage; HSD = human sperm DNA; ICMD = inadequate cellular material for diagnosis; MSP = methylation-specific polymerase chain reaction; NA = nipple aspiration; PCR = polymerase chain reaction.

## Competing interests

The authors declare that they have no competing interests.

## Authors' contributions

IL and RAE conceived of the study. IL conducted the methylation analysis, performed the statistical analysis and drafted the manuscript. ZK-J participated in the design of the study, assisted in performing the methylation analysis and statistical analysis and helped draft the manuscript. MJF designed the methodology of the methylation analysis and helped draft the manuscript. EB participated in the design and coordination of the study and helped draft the manuscript. PO and AN performed the cytological analysis. LI, GP, GPHG and RAE participated in the design and coordination of the study and helped draft the manuscript. All authors read and approved the final manuscript.

## Figures and Tables

**Table 1:** Frequency of hypermethylated genes in *BRCA *gene mutation carriers and controls

Group	*n*	Frequency of hypermethylated genes (percentage)			
		
		*HIN-1*	*RAR-β*	*Twist*	*Cyclin D2*
*BRCA *carriers	19	5 (26)	4 (21)	3 (16)	1 (5)
Controls	5	0 (0)	0 (0)	0 (0)	0 (0)

## References

[B1] Ford D, Easton DF, Stratton M, Narod S, Goldgar D, Devilee P, Bishop DT, Weber B, Lenoir G, Chang-Claude J (1998). Genetic heterogeneity and penetrance analysis of the BRCA1 and BRCA2 genes in breast cancer families. The Breast Cancer Linkage Consortium. Am J Hum Genet.

[B2] Wooster R, Weber BL (2003). Breast and ovarian cancer. N Engl J Med.

[B3] Buist DS, Porter PL, Lehman C, Taplin SH, White E (2004). Factors contributing to mammography failure in women aged 40 – 49 years. J Natl Cancer Inst.

[B4] Leach MO, Boggis CR, Dixon AK, Easton DF, Eeles RA, Evans DG, Gilbert FJ, Griebsch I, Hoff RJ, Kessar P (2005). Screening with magnetic resonance imaging and mammography of a UK population at high familial risk of breast cancer: a prospective multicentre cohort study (MARIBS). Lancet.

[B5] Dooley WC, Ljung BM, Veronesi U, Cazzaniga M, Elledge RM, O'Shaughnessy JA, Kuerer HM, Hung DT, Khan SA, Phillips RF (2001). Ductal lavage for detection of cellular atypia in women at high risk for breast cancer. J Natl Cancer Inst.

[B6] Jones PA, Baylin SB (2002). The fundamental role of epigenetic events in cancer. Nat Rev Genet.

[B7] Esteller M, Silva JM, Dominguez G, Bonilla F, Matias-Guiu X, Lerma E, Bussaglia E, Prat J, Harkes IC, Repasky EA (2000). Promoter hypermethylation and BRCA1 inactivation in sporadic breast and ovarian tumors. J Natl Cancer Inst.

[B8] Ottaviano YL, Issa JP, Parl FF, Smith HS, Baylin SB, Davidson NE (1994). Methylation of the estrogen receptor gene CpG island marks loss of estrogen receptor expression in human breast cancer cells. Cancer Res.

[B9] Widschwendter M, Berger J, Hermann M, Muller HM, Amberger A, Zeschnigk M, Widschwendter A, Abendstein B, Zeimet AG, Daxenbichler G (2000). Methylation and silencing of the retinoic acid receptor-beta2 gene in breast cancer. J Natl Cancer Inst.

[B10] Fackler MJ, McVeigh M, Evron E, Garrett E, Mehrotra J, Polyak K, Sukumar S, Argani P (2003). DNA methylation of RASSF1A, HIN-1, RAR-β, Cyclin D2 and Twist in *in situ *and invasive lobular breast carcinoma. Int J Cancer.

[B11] Evron E, Umbricht CB, Korz D, Raman V, Loeb DM, Niranjan B, Buluwela L, Weitzman SA, Marks J, Sukumar S (2001). Loss of cyclin D2 expression in the majority of breast cancers is associated with promoter hypermethylation. Cancer Res.

[B12] Herman JG, Merlo A, Mao L, Lapidus RG, Issa JP, Davidson NE, Sidransky D, Baylin SB (1995). Inactivation of the CDKN2/p16/MTS1 gene is frequently associated with aberrant DNA methylation in all common human cancers. Cancer Res.

[B13] Graff JR, Herman JG, Lapidus RG, Chopra H, Xu R, Jarrard DF, Isaacs WB, Pitha PM, Davidson NE, Baylin SB (1995). E-cadherin expression is silenced by DNA hypermethylation in human breast and prostate carcinomas. Cancer Res.

[B14] Raman V, Martensen SA, Reisman D, Evron E, Odenwald WF, Jaffee E, Marks J, Sukumar S (2000). Compromised HOXA5 function can limit p53 expression in human breast tumours. Nature.

[B15] Leon SA, Shapiro B, Sklaroff DM, Yaros MJ (1977). Free DNA in the serum of cancer patients and the effect of therapy. Cancer Res.

[B16] Chan MW, Chan LW, Tang NL, Tong JH, Lo KW, Lee TL, Cheung HY, Wong WS, Chan PS, Lai FM (2002). Hypermethylation of multiple genes in tumor tissues and voided urine in urinary bladder cancer patients. Clin Cancer Res.

[B17] Rosas SL, Koch W, Costa Carvalho MG, Wu L, Califano J, Westra W, Jen J, Sidransky D (2001). Promoter hypermethylation patterns of p16, O^6^-methylguanine-DNA-methyltransferase, and death-associated protein kinase in tumors and saliva of head and neck cancer patients. Cancer Res.

[B18] Ahrendt SA, Chow JT, Xu LH, Yang SC, Eisenberger CF, Esteller M, Herman JG, Wu L, Decker PA, Jen J (1999). Molecular detection of tumor cells in bronchoalveolar lavage fluid from patients with early stage lung cancer. J Natl Cancer Inst.

[B19] Belinsky SA, Nikula KJ, Palmisano WA, Michels R, Saccomanno G, Gabrielson E, Baylin SB, Herman JG (1998). Aberrant methylation of p16^INK4a ^is an early event in lung cancer and a potential biomarker for early diagnosis. Proc Natl Acad Sci USA.

[B20] Kurakawa E, Shimamoto T, Utsumi K, Hirano T, Kato H, Ohyashiki K (2001). Hypermethylation of p16^INK4a ^and p15^INK4b ^genes in non-small cell lung cancer. Int J Oncol.

[B21] Suh CI, Shanafelt T, May DJ, Shroyer KR, Bobak JB, Crawford ED, Miller GJ, Markham N, Glode LM (2000). Comparison of telomerase activity and GSTP1 promoter methylation in ejaculate as potential screening tests for prostate cancer. Mol Cell Probes.

[B22] Evron E, Dooley WC, Umbricht CB, Rosenthal D, Sacchi N, Gabrielson E, Soito AB, Hung DT, Ljung B, Davidson NE (2001). Detection of breast cancer cells in ductal lavage fluid by methylation-specific PCR. Lancet.

[B23] Krassenstein R, Sauter E, Dulaimi E, Battagli C, Ehya H, Klein-Szanto A, Cairns P (2004). Detection of breast cancer in nipple aspirate fluid by CpG island hypermethylation. Clin Cancer Res.

[B24] Herman JG, Graff JR, Myohanen S, Nelkin BD, Baylin SB (1996). Methylation-specific PCR: a novel PCR assay for methylation status of CpG islands. Proc Natl Acad Sci USA.

[B25] Fackler MJ, McVeigh M, Mehrotra J, Blum MA, Lange J, Lapides A, Garrett E, Argani P, Sukumar S (2004). Quantitative multiplex methylation-specific PCR assay for the detection of promoter hypermethylation in multiple genes in breast cancer. Cancer Res.

[B26] Wrensch MR, Petrakis NL, Miike R, King EB, Chew K, Neuhaus J, Lee MM, Rhys M (2001). Breast cancer risk in women with abnormal cytology in nipple aspirates of breast fluid. J Natl Cancer Inst.

[B27] Kurian AW, Mills MA, Jaffee M, Sigal BM, Chun NM, Kingham KE, Collins LC, Nowels KW, Plevritis SK, Garber JE (2005). Ductal lavage of fluid-yielding and non-fluid-yielding ducts in BRCA1 and BRCA2 mutation carriers and other women at high inherited breast cancer risk. Cancer Epidemiol Biomarkers Prev.

[B28] Mitchell G, Antill YC, Murray W, Kirk J, Salisbury E, Lindeman GJ, Di Iulio J, Milner AD, Devereaux L, Phillips KA (2005). Nipple aspiration and ductal lavage in women with a germline BRCA1 or BRCA2 mutation. Breast Cancer Res.

[B29] Maddux AJ, Ashfaq R, Naftalis E, Leitch AM, Hoover S, Euhus D (2004). Patient and duct selection for nipple duct lavage. Am J Surg.

[B30] Isaacs C, Cavalli LR, Cohen Y, Pennanen M, Shankar LK, Freedman M, Singh B, Liu M, Gallagher A, Rone JD (2004). Detection of LOH and mitochondrial DNA alterations in ductal lavage and nipple aspirate fluids from hngh-risk patients. Breast Cancer Res Treat.

[B31] King BL, Tsai SC, Gryga ME, D'Aquila TG, Seelig SA, Morrison LE, Jacobson KK, Legator MS, Ward DC, Rimm DL (2003). Detection of chromosomal instability in paired breast surgery and ductal lavage specimens by interphase fluorescence *in situ *hybridization. Clin Cancer Res.

[B32] Fackler MJ, Malone K, Zhang Z, Schilling E, Garrett-Mayer E, Swift-Scanlan T, Lange J, Nayar R, Davidson NE, Khan SA (2006). Quantitative multiplex methylation-specific PCR analysis doubles detection of tumor cells in breast ductal fluid. Clin Cancer Res.

[B33] Khan SA, Wiley EL, Rodriguez N, Baird C, Ramakrishnan R, Nayar R, Bryk M, Bethke KB, Staradub VL, Wolfman J (2004). Ductal lavage findings in women with known breast cancer undergoing mastectomy. J Natl Cancer Inst.

[B34] Higgins SA, Matloff ET, Rimm DL, Dziura J, Haffty BG, King BL (2005). Patterns of reduced nipple aspirate fluid production and ductal lavage cellularity in women at high risk for breast cancer. Breast Cancer Res.

[B35] Locke I, Kote-Jarai Z, Bancroft E, Bullock S, Jugurnauth S, Osin P, Nerurkar A, Izatt L, Pichert G, Gui GPH (2006). LOH at the *BRCA1 *and *BRCA2 *loci detected in ductal lavage fluid from *BRCA *gene mutation carriers and controls. Cancer Epidemiol Biomarkers Prev.

[B36] Antill YC, Mitchell G, Johnson SA, Devereux L, Milner A, Phillips KA, Campbell IG (2006). Loss of heterozygosity analysis in ductal lavage samples from BRCA1 and BRCA2 carriers: a cautionary tale. Cancer Epidemiol Biomarkers Prev.

[B37] Fraker LD, Halter SA, Forbes JT (1984). Growth inhibition by retinol of a human breast carcinoma cell line *in vitro *and in athymic mice. Cancer Res.

[B38] Sirchia SM, Ferguson AT, Sironi E, Subramanyan S, Orlandi R, Sukumar S, Sacchi N (2000). Evidence of epigenetic changes affecting the chromatin state of the retinoic acid receptor β2 promoter in breast cancer cells. Oncogene.

[B39] Krop IE, Sgroi D, Porter DA, Lunetta KL, LeVangie R, Seth P, Kaelin CM, Rhei E, Bosenberg M, Schnitt S (2001). HIN-1, a putative cytokine highly expressed in normal but not cancerous mammary epithelial cells. Proc Natl Acad Sci USA.

[B40] Widschwendter M, Jones PA (2002). DNA methylation and breast carcinogenesis. Oncogene.

